# Chemical Dispersant Enhances Microbial Exopolymer (EPS) Production and Formation of Marine Oil/Dispersant Snow in Surface Waters of the Subarctic Northeast Atlantic

**DOI:** 10.3389/fmicb.2019.00553

**Published:** 2019-03-20

**Authors:** Laura Duran Suja, Xindi Chen, Stephen Summers, David M. Paterson, Tony Gutierrez

**Affiliations:** ^1^School of Engineering and Physical Sciences, Heriot-Watt University, Edinburgh, United Kingdom; ^2^School of Biology, Scottish Oceans Institute, University of St Andrews, St Andrews, United Kingdom; ^3^College of Harbour, Coastal and Offshore Engineering, Hohai University, Nanjing, China; ^4^Singapore Centre for Environmental Life Sciences Engineering, Nanyang Technological University, Singapore, Singapore

**Keywords:** marine oil snow, marine dispersant snow, Faroe–Shetland Channel, hydrocarbon-degrading bacteria, EPS

## Abstract

A notable feature of the Deepwater Horizon oil spill was the unprecedented formation of marine oil snow (MOS) that was observed in large quantities floating on the sea surface and that subsequently sedimented to the seafloor. Whilst the physical and chemical processes involved in MOS formation remain unclear, some studies have shown that extracellular polymeric substances (EPS) play a role in this process. Here, we report that during exposure of subarctic northeast Atlantic seawater to a chemical dispersant, whether in the presence/absence of crude oil, the dispersant stimulates the production of significant quantities of EPS that we posit serves as a key building block in the formation of MOS. This response is likely conferred via *de novo* synthesis of EPS by natural communities of bacteria. We also describe the formation of marine dispersant snow (MDS) as a product of adding chemical dispersants to seawater. Differential staining confirmed that MDS, like MOS, is composed of glycoprotein, though MDS is more protein rich. Using barcoded-amplicon Illumina MiSeq sequencing, we analyzed, for the first time, the bacterial communities associated with MDS and report that their diversity is not significantly dissimilar to those associated with MOS aggregates. Our findings emphasize the need to conduct further work on the effects of dispersants when applied to oil spills at sea, particularly at different sites, and to determine how the product of this (i.e., MOS and MDS) affects the biodegradation of the oil.

## Introduction

During the Deepwater Horizon (DwH) oil spill, which occurred in the Gulf of Mexico on April 20 of 2010, oil-associated marine snow, referred to as marine oil snow (MOS), was observed within 2 weeks from the onset of the spill. The spill was unprecedented based on the depth at which it occurred (1500 m below the sea surface), the volume of oil released and dispersant used (respectively, 4.9 million barrels and 2.1 million gallons), and the formation of large quantities of MOS and its subsequent sedimentation to the seafloor (Brooks et al., 2015; Romero et al., 2015; Daly et al., 2016; Passow, 2016). Evidence of MOS formation has been reported for other major oil spills – namely the Ixtoc-I (Boehm and Fiest, 1980; Jernelöv and Lindén, 1981; Patton et al., 1981) and *Tsesis* (Johansson et al., 1980) oil spills. Like for marine snow, MOS is defined as oil-entrained mucilaginous flocs or particles ranging from >0.5 mm to 10s of centimeters in size, and composed of organic (e.g., exopolymeric) and inorganic (e.g., mineral) substances, microorganisms (e.g., bacterial and micro-algal cells), and other biogenic and inert components in seawater (Simon et al., 2002; Passow et al., 2012; Fu et al., 2014; Daly et al., 2016). Whilst MOS formation may be a product of the interaction between suspended organic matter and oil (Fu et al., 2014; Kleindienst et al., 2015), the underlying mechanism(s) in this process have yet to be fully understood. Various factors, such as hydrodynamic conditions, collision rate of suspended particles, particle coagulation and flocculation, and the interaction of particles with oil components/droplets, as well as with microorganisms and their produced exopolymers, are considered important in this process (Passow et al., 2012; Daly et al., 2016).

A wide variety of microorganisms, particularly cyanobacteria (Kawaguchi and Decho, 2002; Decho et al., 2005; Han et al., 2014), bacteria (Kennedy and Sutherland, 1987; Grossart et al., 2007; Thavasi and Banat, 2014) and eukaryotic phytoplankton (Myklestad, 1995, 1977; Mishra and Jha, 2009; Raposo et al., 2013) produce and secrete exopolysaccharide substances (EPS) in the marine environment and which contributes significantly to the dissolved organic carbon (DOC) in the global ocean water column – ca. 10–25% of total oceanic dissolved organic matter (Verdugo, 1994; Aluwihare et al., 1997). Compared to EPS produced by freshwater/marine eukaryotic phytoplankton and non-marine bacteria, EPS produced by marine bacteria generally contain higher levels of uronic acids, notably D-glucuronic and D-galacturonic acids (Kennedy and Sutherland, 1987). These acidic sugars can render these macromolecules polyanionic (negatively charged) and “sticky,” and consequently confer them with the ability to form aggregates, such as marine snow and transparent exopolymer particles (TEP) (Wotton, 2004a). Hence, a significant proportion of the EPS in the ocean is in the form of acidic polysaccharides (APS), which are found in dissolved form and as a major component of TEP, as well as involved as a protective external layer of some microorganisms (Thornton, 2009). As one of the most common types of EPS produced by bacteria and eukaryotic phytoplankton (Passow and Alldredge, 1994; Stoderegger and Herndl, 1999), APS is known to contribute to the formation of marine snow (Alldredge et al., 1993), and recent work has shown it to be implicated also in the formation of MOS (Passow et al., 2012; Gutierrez et al., 2013, 2018; Passow, 2016).

Unprecedented quantities, up to 7 million liters, of the dispersant Corexit EC9500A was applied by spraying on sea surface oil slicks and subsequently directly injected at the leaking wellhead near the seafloor during the DwH incident (NatioNational Commission on the Bp Deepwater Horizon Oil Spill and Offshore Drillingal, 2011); this was after the dispersant Corexit 9527 was used initially. This surface and subsurface application of Corexit was reported to result in facilitated microbial biodegradation of the oil (Brakstad et al., 2015), whilst a study by Kleindienst et al. (2015) showed that certain members of the hydrocarbon-degrading community (specifically *Marinobacter*) in the Gulf of Mexico could be inhibited by Corexit application. Whilst chemical dispersants, including Corexit, have been reported to trigger or enhance MOS formation (Baelum et al., 2012; Fu et al., 2014; Kleindienst et al., 2015; Passow, 2016; Suja et al., 2017), the mechanisms underlying this process remain unclear. An intriguing observation in some of these studies has been the formation of flocs/aggregates when only Corexit, but no oil, was present (Kleindienst et al., 2015; Suja et al., 2017). These flocs/aggregates, which appear white to off-white in coloration, are quite “sticky” or gelatinous when handled, and whilst they can be defined as a type of marine snow, they have received very little attention. In the event of an oil spill at sea, the surface or subsurface spraying/injection of dispersants is likely to result in areas on the sea surface, or “pockets” within the water column, where dispersant molecules would not directly interact with the oil. Free dispersant molecules would likely interact with DOC, as well as microbial cells, leading to the formation of MDS. The composition, fate and impact of MDS in the marine environment, however, remains largely unexplored. To-date, observations of MDS have only been documented in two reports employing laboratory-based experiments (Kleindienst et al., 2015; Suja et al., 2017); hence, their potential to form during the application of dispersants at sea during an oil spill warrants attention. Whilst the mechanism(s) involved in MOS formation still remain largely unresolved, the prevailing evidence implicates EPS and dispersants, either independently or in combination, as key agents in this process. Here, we investigated the role of EPS (APS as proxy) in MOS formation in surface waters of a subarctic region in the northeast Atlantic, and how this process might be influenced by chemical dispersants. We also assess MDS formation and analyze the communities of bacteria associated with these particles and compare them to those with MOS.

## Materials and Methods

### Field Samples

During a research cruise on RV *Scotia* on October 11, 2017, sea surface water samples were collected from a depth of 5 m at a subarctic northeast Atlantic region called the Faroe–Shetland Channel (FSC) (60°38.120′ N, 4°54.030′ W) – *in situ* temp. 8.7°C. This sampling site was ca. 10 km from the Schiehallion oilfield and located along the Fair Isle-Munken (FIM) line, which is a sampling transect that runs between the Faroe and Shetland Isles, north of Scotland. The surface water of this region is defined as a water mass that is relatively warmer and saltier as compared to the underlying Arctic/Icelandic cold-water masses that are found at depths from 400 to 1500 m in this region (Berx et al., 2013). The water samples were immediately stored at 4°C aboard the ship in 10 L carboys and used within 1 week for the preparation of water-accommodated fractions (WAFs) and enrichment experiments with crude oil, dispersant and/or nutrient amendment, as described below.

### Water-Accommodated Fractions

A water accommodated fraction (WAF) is defined as a laboratory-based medium containing dispersed and solubilized crude oil hydrocarbons. It is produced by mixing seawater with crude oil, and subsequent removal of the non-dispersed/non-solubilized oil. Here, WAF was prepared following the method of Kleindienst et al. (2015), with the exception that seawater filtrates were not pasteurized as we did not want to potentially alter the chemistry of the seawater, or properties of endogenous EPS, by a heat treatment step. Briefly, sea surface water collected from the FSC was filtered through 0.22 μm filters to remove microbial cells. A 800 mL volume of filtered seawater was amended with 140.8 mL of pre-filtered (0.22 μm) Schiehallion crude oil (provided by BP). To prepare chemically enhanced WAF (CEWAF) medium, 800 mL of filtered seawater was amended with 140.8 mL of filtered Schiehallion crude oil and 14.08 mL of Superdispersant-25 (provided by Oil Slick Dispersants Ltd.). Seawater amended with only dispersant comprised 800 mL of the filter-sterile seawater and 14.08 mL of Superdispersant-25. The effective dilution of the dispersant in the seawater treatments (dispersant-to-seawater ratio, v/v) was 1:10, which is a dilution recommended by the oil and gas industry (Approved oil spill treatment products, UK Government, July 2016).

The various mixtures of sterile seawater (SW) amended with oil (WAF), oil+dispersant (CEWAF) or solely dispersant (SW+D) were mixed on a rotary magnetic stirrer at 140 rpm for 48 h at 7°C in the dark in clean sterile (acid-washed) 1 L glass bottles. The mixtures were then allowed to stand for 1 h prior to transferring the aqueous phases into clean Teflon-lined screw-capped glass tubes whilst avoiding the non-dispersed/non-solubilized oil. The SW and these WAF, CEWAF and SW+D solutions were then stored at 4°C and used within 48 h for the microcosm experiments. For treatments containing nutrients – i.e., seawater+nutrients (SW+N) and CEWAF+nutrients (CEWAF+N) – the solutions were amended with 10 μM ammonium chloride, 10 μM sodium nitrate and 1 μM potassium phosphate (final concentrations).

### Microcosm Setup and Sampling

To examine the microbial response and formation of MOS and MDS in sea surface waters of the FSC when exposed to crude oil, dispersant and/or nutrients, a roller-bottle design was used, as previously described (Suja et al., 2017). This roller bottle setup has been used widely to investigate marine snow (Shanks and Edmondson, 1989) and MOS (Kleindienst et al., 2015; Passow, 2016) formation as it simulates natural sea surface/pelagic conditions in a laboratory setting (Jackson, 1994). For this experiment, four microcosm treatments (WAF, SW+D, CEWAF, CEWAF+N) were setup, each prepared using 500 mL Pyrex glass bottles that were maintained at constant rotating gentle motion. Each treatment was run in triplicate and comprised of 85.5 mL of filter-sterile WAF, dispersant-only, CEWAF or CEWAF+N added to 300 mL of unfiltered natural seawater from the FSC. In addition, two oil/dispersant untreated controls were setup and run in parallel: one comprised seawater alone (SW), and the second of seawater with only nutrients added (SW+N). Treatments and controls were each established in triplicates and incubated at 8°C (*in situ* sea surface temp. in the FSC at the time of sampling) and in the dark at a rotation speed of 15 rpm.

All bottles for each treatment and the controls were sampled, taking extreme care in order to avoid disrupting aggregates that may have formed. Sampling was performed at five time points over the course of 4 weeks: at the beginning of the experiment (T0), after 1 week (T1), after 2 weeks (T2), after 3 weeks (T3), and after 4 weeks (T4). At each sampling time, each bottle was placed gently in an upright position to capture a photographic record of the contents within (e.g., change in color and formation of MOS or MDS). When formed, sub-samples of MOS or MDS aggregates were carefully withdrawn using pre-sterilized glass Pasteur pipettes and transferred to 1.5-mL microcentrifuge tubes for staining with the cationic copper phthalocyanine dye alcian blue (AB) at pH 2.5 (Alldredge et al., 1993), or with the amino acid-specific dye coomassie brilliant blue (CBBG) at pH 7.4 (Long and Azam, 1996). AB is used for staining acidic sugars of EPS or TEP in seawater, whereas CBBG is used for staining the proteinaceous component of these polymeric substances. Following staining, the aggregates were washed by transferring them through several droplets of sterile water prior to their examination under the light microscope. To directly examine the prokaryotic community under the microscope, MOS and MDS particles were also stained with acridine orange (AO) (Francisco et al., 1973) for imaging with a FITC filter on a Zeiss Axioscope epifluorescence microscopy (Carl Zeiss, Germany). Moreover, MOS or MDS aggregates were also sub-sampled during these experiments for DNA extraction and analysis of their associated bacterial community (described below). Observations of all treatments and controls were also recorded for changes in turbidity and/or emulsion formation.

### Genomic DNA Extraction and Barcoded-Amplicon Sequencing and Analysis

DNA was extracted from MDS and MOS aggregates according to the method of Tillett and Neilan (2000). Purified DNA was stored at -20°C for subsequent molecular analysis. Barcoded 16S rRNA gene MiSeq sequencing, targeting the V4 hypervariable region, was employed to analyze the bacterial community of the different aggregates over the 4-week duration of the experiments at time points T1, T2, T3, and T4. We amplified the 16S rRNA gene in duplicate 25 μl reactions, and replicates were subsequently pooled to increase PCR product yield. Each reaction comprised 10.5 μl molecular biology grade water, 12.5 μl of Platinum Hot Start Master Mix, 0.5 μl each of 10 μM forward (515f) and reverse (806r) primers, and 1 μl of template DNA. The primers used were 515f (GTGCCAGCMGCCGCGGTAA) and 806r (GGACTACNVGGGTWTCTAAT) (Caporaso et al., 2011, 2012). Both primers had Illumina MiSeq overhangs and unique golay barcodes added to the 5′ ends. All PCR products were purified by GFX PCR purification (#GE28-9034-70, Sigma, United Kingdom). All samples were sequenced via the Illumina MiSeq platform (Illumina 2 × 250 V.2 kit) at the Edinburgh Genomics sequencing facility (University of Edinburgh, United Kingdom), and raw sequences were demultiplexed prior to receipt at our laboratory. All sequences were deposited in the SRA repository under accession number SAMN10417097 to SAMN10417120.

Subsequent processing of the Illumina sequence data was performed using the DADA2 package as wrapped in QIIME2 (Callahan et al., 2016). In brief, paired end Illumina reads (Phred 33) were combined to form contiguous sequences. A fragment cut-off of 220 bp was established to maintain quality. These contigs were examined for low quality phred scores and any identified chimeric sequences were removed. All quality-approved sequences were compared on a single nucleotide resolution and the resulting single nucleotide variants (SNVs) were identified using the Green Genes database of 16S rRNA gene taxonomy.

*Alpha*- and *beta*-diversity indices were collected for the individual samples and treatment types, respectively. For *alpha*-diversity analysis, sampling was standardized to a ceiling of 44,800 sequences per sample, and rarefaction and ordination analyses both utilized this standardization. All sequences were deposited in the SRA repository under accession number SAMN10417097 to SAMN10417120.

### EPS Extraction and Analysis

Alcian blue is a cationic dye which binds with the carboxyl (COO^-^) and half-ester sulfate (-OSO_3_^-^) groups of APS, but does not complex with neutral sugars (Ramus, 1977; Passow and Alldredge, 1995). Hence, it was used here to quantify APS as a proxy of EPS concentrations in the microcosm treatments. For this, a stock solution of Alcian Blue (GX8) was prepared (0.02% w/v final concentration) in distilled water containing 0.06% v/v acetic acid (analytical grade). The solution was sonicated for 15 min to disaggregate particulates of the Alcian Blue, then stirred for 30 min with a magnetic stirrer, and the solution stored at 4°C. Prior to use, the Alcian Blue solution was several times passed through 0.45 μm filters to remove any dye coagulation. The stability of the final solution was determined by a constant absorbance reading (602 nm) between sequential filtrations. A standard curve was produced using xanthan gum as a proxy for natural APS (after Hope, 2016) and total APS concentrations were obtained as xanthan equivalents (μg X eq. mL^-1^).

Aliquots for APS analysis (1.5 ml) were transferred to 3 mL glass centrifuge tubes. The samples were vortexed for 30 s, centrifuged (2500 rpm; 20 min), and then 1 mL of pre-filtered Alcian blue solution added to each sample. The Alcian-sample mixture was resuspended and vortexed for 30 s to ensure an irreversible bond formed between the Alcian blue and APS. The samples were then centrifuged (2500 rpm; 20 min) to release unbound dye into the supernatant, which was carefully removed. The remaining dyed pellet was rinsed with distilled water and centrifuged again, and this was repeated until no excess dye was released (cf. 3 washes). The remaining stained pellets were treated with 6 mL of 80% (v/v) sulphuric acid, suspended and sonicated for 15 min and then vortexed for 30 s to obtain a homogenous solution. The samples were gently agitated 2–3 times during an incubation period of 2 h (color stable for 2–20 h), to release any oxygen bubbles. Sulphuric acid disassociated the bound dye from the pellet, resulting in a gradation of green solutions depending on the concentration of APS in the samples. The absorbance of each sample was measured spectrophotometrically (787 nm, Biomate 5 spectrophotometer).

### Statistical Analyses

Relative abundances of sequences obtained using MiSeq were compared between treatments by PERMANOVA, at a level 5 taxonomy (family). EPS concentrations, based on APS measurements, were compared between the different treatments and timeline using Analysis of Variance (ANOVA) followed by a *post hoc* Tukey test.

## Results and Discussion

### Formation and Chemical Composition of MOS

**FIGURE 1:**
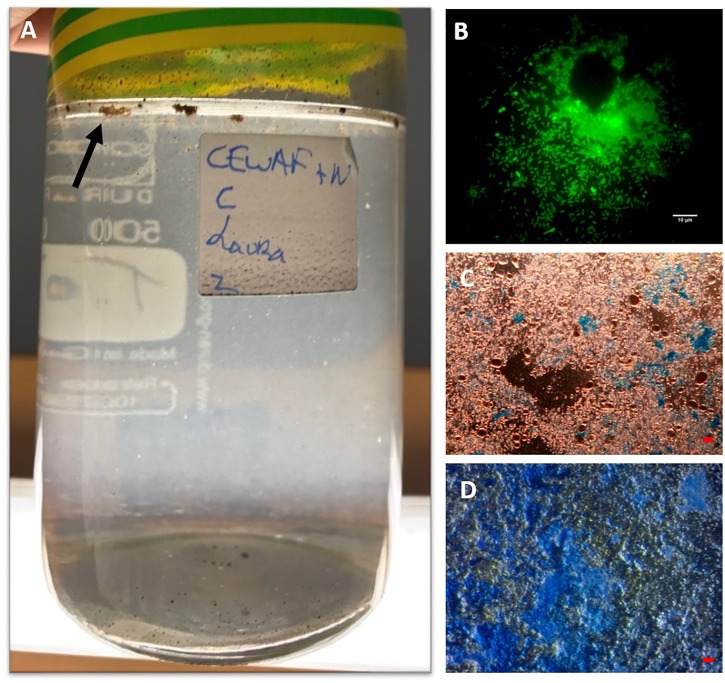
MOS aggregates formed in the CEWAF+N treatment shown at 4 weeks floating on the surface (indicated by arrow) of the roller bottle **(A)**. An aggregate from this treatment stained with acridine orange and viewed under the epifluorenscence microscope with a FITC filter shows a rich community of prokaryotic cells (small green dots) **(B)**. MOS stained with coomassie brilliant blue G **(C)**, and Alcian Blue **(D)**. Bar, 10 μm.

The formation of MOS was observed in the CEWAF and CEWAF+N treatments within 6 days since the commencement of these experiments. Initially, the MOS particles appeared small (0.5–1 mm) and dark brown, and by the end of these incubations (at day 28) they had increased in size (2–5 mm) and remained floating at the surface (Figure 1A). They did not settle to the bottom of the bottles at any time over the 4-week duration of these experiments. At the termination of these experiments (T4), the particles appeared somewhat lighter in color, suggesting that the oil hydrocarbons entrained within the MOS particles had become, at least partially, degraded. As previously reported by our group (Suja et al., 2017), the presence of the chemical dispersant Superdispersant-25 was a key component to yielding MOS, and the presence of nutrients magnified this response – i.e., the abundance of MOS particles in the CEWAF+N treatment was much higher than in the CEWAF treatment without nutrients. MOS also formed in the CEWAF treatment, but because they were comparatively smaller in size and consequently difficult to handle, they were not further analyzed. We note that this dispersant-induced formation of MOS is reproducible across seasons of the year, as this response was demonstrated in roller bottle experiments performed at our laboratory using surface seawater collected from the FSC in the winter of 2015 (Suja et al., 2017). Conversely, we did not observe the formation of MOS in roller bottle experiments using surface seawater collected from the FSC during the spring of 2015 when exposed to solely Schiehallion crude oil and without chemical dispersants (results not shown). Corroborating with our previous results (Suja et al., 2017), the formation of MOS was not observed in the WAF treatment (no dispersant added), and similarly we observed the formation of marine snow (without oil) in the SW+N treatment.

These results add to a growing body of evidence indicating that in the event of an oil spill at sea, the application of chemical dispersants is important in triggering the formation of MOS (Fu et al., 2014; Kleindienst et al., 2015; Suja et al., 2017; Doyle et al., 2018). However, it should be noted that under certain conditions, which remain unresolved, MOS can form in the absence of a chemical dispersant. Passow et al. (2017), for example, showed that diatoms resulted in increased MOS formation, whereas its formation in the presence of diatoms was inhibited when dispersant was added. In the absence of dispersant application for any specific oceanic region, any number of environmental factors (e.g., sea surface and hydrodynamic variables, algal/bacterial community profile, DOC/EPS concentrations in seawater etc.) could be implicated to initiating MOS during an oil spill. Our results show that in surface waters of the FSC – a subarctic region of the northeast Atlantic – the application of a chemical dispersant does result in the formation of MOS and MDS.

When observed under the epifluorescence microscope with AO staining, MOS aggregates from the CEWAF and CEWAF+N treatments were heavily enriched with prokaryotic cells (Figure 1B) – some aggregates were also observed to contain oil droplets (not shown). When viewed under the light microscope with the aid of dark field illumination, these aggregates appeared to partially stain with CBBG (Figure 1C), whereas they predominately stained with AB (Figure 1D). This provides evidence that MOS aggregates formed in the presence of the dispersant, oil and with/without nutrients (i.e., the CEWAF and CEWAF+N treatments) were of glycoprotein composition, with a heavy compositional loading of EPS. Previous work assessing MOS formation in waters of the Gulf of Mexico during the active phase of the DwH oil spill showed MOS aggregates as highly susceptible to peptidase and β-glucosidase activities (Ziervogel et al., 2012), which was indicative of their glycoprotein composition. As glycoproteins are a major component of marine bacterial EPS (Long and Azam, 1996; Mancuso Nichols et al., 2004; Verdugo et al., 2004; Hassler et al., 2011), these studies collective point to marine bacteria as a source of these polymers and in playing a key role in MOS formation. The amino acid and peptide components comprising these bacterial glycoproteins can confer amphiphilic characteristics to these macromolecules (Verdugo et al., 2004; Gutierrez et al., 2009) and in turn allow them to associate with petrochemical hydrocarbons and crude oil droplets.

Previous work assessing MOS formation in surface water samples collected from the Gulf of Mexico well after the DwH spill showed no formation of MOS (Ziervogel et al., 2014). These experiments had been performed without any added chemical dispersant. It can be conjectured, therefore, that by September 2012 (ca. 2 years and 5 months after the onset of the DwH spill when these water samples were collected) there was either none, or insignificant concentrations, of the Corexit remaining in the Gulf water column. MOS, however, formed in roller bottle experiments performed by the same researchers when using seawater collected from the Gulf during the active phase of the DwH spill (Ziervogel et al., 2012). Although no chemical dispersant had been added to those experiments, we suspect that the seawater samples used by Ziervogel et al. (2012) contained Corexit since it had been applied in copious quantities on the sea surface and subsurface, and which would explain the observed formation of MOS and also supporting the role of chemical dispersants in this process. Taken collectively, these and other studies point to chemical dispersants as an important contributor to triggering MOS formation, and that nutrients enhance this response.

### Formation and Chemical Composition of MDS

**FIGURE 2:**
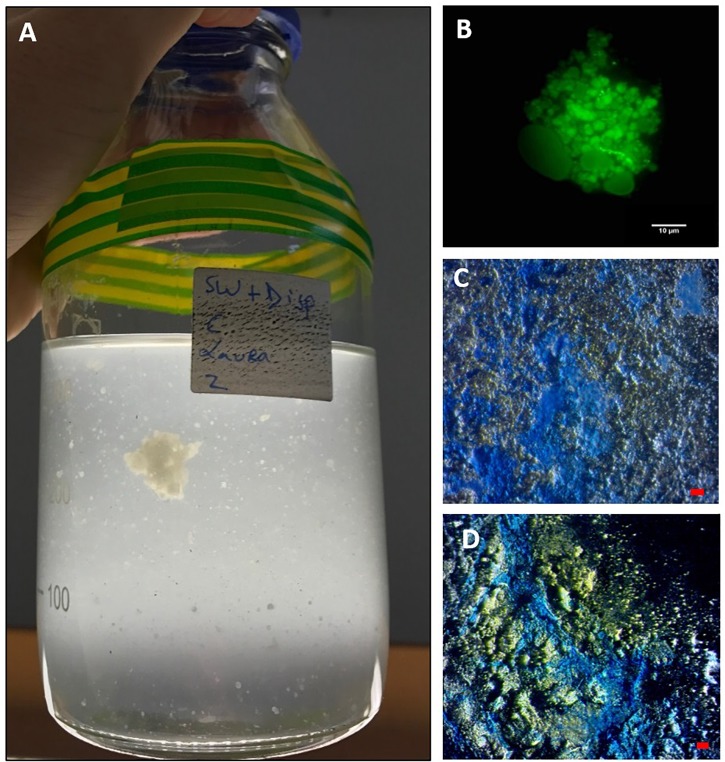
MDS aggregates formed in the SW+D treatment shown at 4 weeks **(A)**. An aggregate from this treatment stained with acridine orange and viewed under the epifluorescence microscope with a FITC filter shows very few associated prokaryotic cells (small green dots), but an apparent abundance of dispersant globules/droplets **(B)**. MDS stained with coomassie brilliant blue G **(C)** and Alcian Blue **(D)**. Bar, 10 μm.

Marine dispersant snow formed within 3 days in the SW+D treatment only, appearing like “cotton wool” and white to off-white in coloration. Over time, some of the particles grew in size (up to 0.5–1.5 mm by the end of the experiment), although many remained quite small (typically < 3 mm) (Figure 2A). When handled, MDS displayed a very “gluey” or “sticky” consistency that was atypical of MOS, but resembled MDS particles that formed in experiments with surface seawater from the Gulf of Mexico amended with Macondo oil and Corexit (Kleindienst et al., 2015), and with seawater collected from the FSC in the spring of 2017 when amended with Scheihallion crude oil and Superdispersant-25 (Suja et al., 2017). Over time, we observed the MDS particles to lose their buoyancy and eventually settle to the bottom of the bottles. This further supports the role of dispersants in MOSSFA (MOS sedimentation and flocculent accumulation), as observed during the DwH spill, by way of initiating the process for the transportation of the oil to the seafloor. We posit that a MOSSFA event could occur in the FSC should chemical dispersants be used in the event of an oil spill in this region, and potentially elsewhere in the northeast Atlantic.

When observed under the epifluorescence microscope with AO staining, MDS particles appeared heavily entrained with droplets that we suspect were composed of Superdispersant-25 due to their partial autofluorescence, since one of the major components of this chemical formula, dioctyl sulphosuccinate (DOSS), exhibits a fluorescence spectrum (e.g., Gray et al., 2014). In contrast to MOS particles (Figure 1B), very few prokaryotic cells were associated with these MDS particles (Figure 2B), which is reminiscent also for the low prokaryotic abundance we have observed associated with marine snow particles formed in our experiments with surface seawater from the FSC (Suja et al., 2017). We are unable, at the present time, to explain this differential cell concentration between MOS and MDS, but we posit that the chemical dispersant may not be a suitable substrate for degradation and assimilation by marine bacteria, and as such these organisms may not find MDS a suitable niche to colonize. When viewed under the light microscope with the aid of dark field illumination, MDS aggregates stained strongly with both CBBG (Figure 2C) and AB (Figure 2D). This indicates that MDS, like MOS, is of glycoprotein composition. However, MDS contained a much higher content of protein, the source of which may, in part, be derived from an endogenous pool of glycoprotein-based EPS in the seawater of the FSC, and more likely via its *de novo* synthesis by communities of EPS-producing bacteria (discussed below).

The formation of MDS in marine waters during the application of chemical dispersants has not yet been directly reported from field observations. When one considers the application of dispersants to an oil spill, such as by aerial spraying onto surface oil slicks or via subsurface injection at or near the source of a leaky well, not all of the dispersant would be expected to directly interact with the oil, but rather diffuse into seawater uncontaminated with oil. In this circumstance, the dispersant will likely follow the same fate in our laboratory-based experiments (Suja et al., 2017 and this study) and those of other researchers (Kleindienst et al., 2015) in forming MDS aggregates that, like MOS, would subsequently fall to the seafloor as defined by the MOSSFA process.

### EPS Response to Dispersant and Crude Oil

**FIGURE 3:**
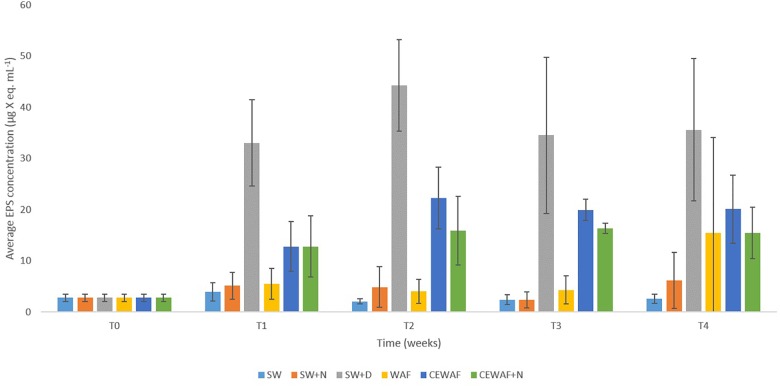
Average EPS concentrations in the different treatments at time points T0 (initial at 0 week), T1 (1 week), T2 (2 weeks), T3 (3 weeks), and T4 (4 weeks). Standard error bars are from triplicate roller bottles for each respective treatment.

Significantly higher concentrations of EPS (PERMANOVA, *p* < 0.05) were detected in treatments amended with the chemical dispersant (SW+D, CEWAF, CEWAF+N) over time compared to treatments with no added dispersant (SW, SW+N, WAF) (Figure 3). Based on APS analysis, average EPS concentrations across all treatments at the start of these experiments (T0) were 2.7 ± 0.7 μg X eq. mL^-1^, which is concomitant with values reported in surface seawater of the subarctic and Arctic (Hong et al., 1997; Ramaiah et al., 2001), and other sites such as the Gulf of Mexico (Thornton et al., 2007). Within 1 week (T1), EPS concentrations increased by ca. 10-fold (to 33.0 ± 8.0 μg X eq. mL^-1^) in the SW+D treatment, and by ca. four-fold (to 13.0 ± 5.0 μg X eq. mL^-1^) in the CEWAF and CEWAF+N treatments. Average concentrations of EPS in these treatments remained high over the remaining duration (weeks 2 to 4) of these experiments, at 33–45 μg X eq. mL^-1^ in the SW+D treatment, and 15–20 μg X eq. mL^-1^ in the CEWAF and CEWAF+N treatments. Preliminary tests to evaluate whether the chemical dispersant Superdispersant-25 could be stained with Alcian blue showed that this to be negative. In contrast, no significant change in EPS concentrations (*p* > 0.05) were detected in the SW, SW+N and WAF treatments (with no added dispersant) over the entire duration of these experiments, with concentrations averaging 3.0–5.5 μg X eq. mL^-1^; the exception was the WAF treatment at week 4, but the increased EPS concentration measured here can be attributed to an outlier of the triplicate samples analyzed, as shown by the large error bar range.

In previous work (Suja et al., 2017) and in this study, we showed that chemical dispersants and nutrients enhance MOS formation, particularly in their size and abundance. Here, we also show that the presence of a chemical dispersant can increase the concentration of EPS in seawater. Similarly, EPS consisting of proteins and polysaccharides was produced in laboratory experiments when exposing eukaryotic phytoplankton communities to chemical dispersants (Van Eenennaam et al., 2016). Notably, in our experiments this phenomenon also occurred in the absence of crude oil (SW+D treatment) where this measured increase in EPS concentration was highest across the various treatments; although it was also significantly higher in the other dispersant-amended treatments (CEWAF, CEWAF+N) compared to the treatments without dispersant. Furthermore, it should also be noted that the presence of nutrients (i.e., in the CEWAF+N treatment) did not yield higher concentrations of EPS, even though this treatment led to the formation of large and more abundant MOS (Suja et al., 2017 and this study).

Our findings show that only in treatments where the chemical dispersant was applied had EPS concentrations significantly increased, and it is in only these treatments where MOS and MDS formed. During an oil spill at sea, bacteria are the major responders, whereas eukaryotic phytoplankton are often susceptible and detrimentally affected by the toxic effects of oil hydrocarbons, as reported for the DwH spill (Parsons et al., 2015). Hence, the dispersant-mediated enhancement in EPS production could largely, if not entirely, be attributed to EPS-producing bacteria that also comprise the community associated with MOS and MDS (discussed below). The dispersant may offer a labile source of carbon to these types of bacteria, which they may metabolize for growth but also convert to EPS. Various lines of speculation could be offered to explain why EPS production is induced in the presence of chemical dispersants, one of which is as a response to stressors (Wotton, 2004a,b), such as exposure to the dispersant itself and in the presence/absence of crude oil (Passow et al., 2012). In this respect, the crude oil and the chemical dispersant in combination (CEWAF or CEWAF+N treatments), or the dispersant alone (SW+D), act as the stressor and yielded heightened levels of EPS. However, crude oil alone cannot be defined as a stressor in this respect because EPS concentrations in the WAF treatment did not significantly increase EPS concentrations compared to the untreated controls (Figure 3).

### Bacterial Community Composition of MOS and MDS

**FIGURE 4:**
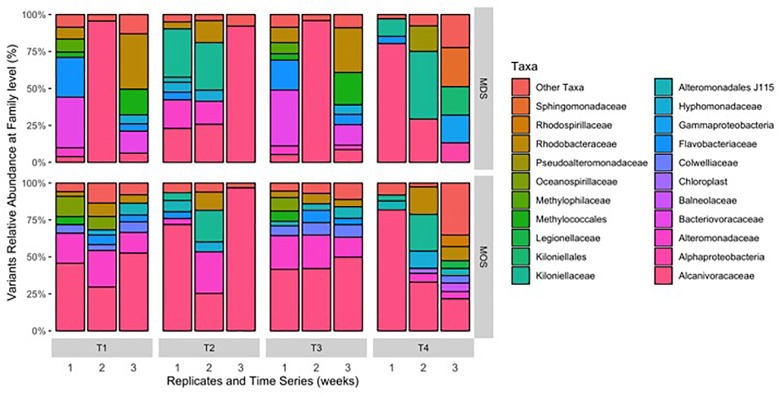
Composition of bacterial 16S rRNA gene MiSeq reads for MDS and MOS aggregates at weeks 1 (T1), 2 (T2), 3 (T3), and 4 (T4) from treatments SW+D and CEWAF+N, respectively. Sequences were classified to family level taxonomy when possible and otherwise at higher level classification is shown.

**FIGURE 5:**
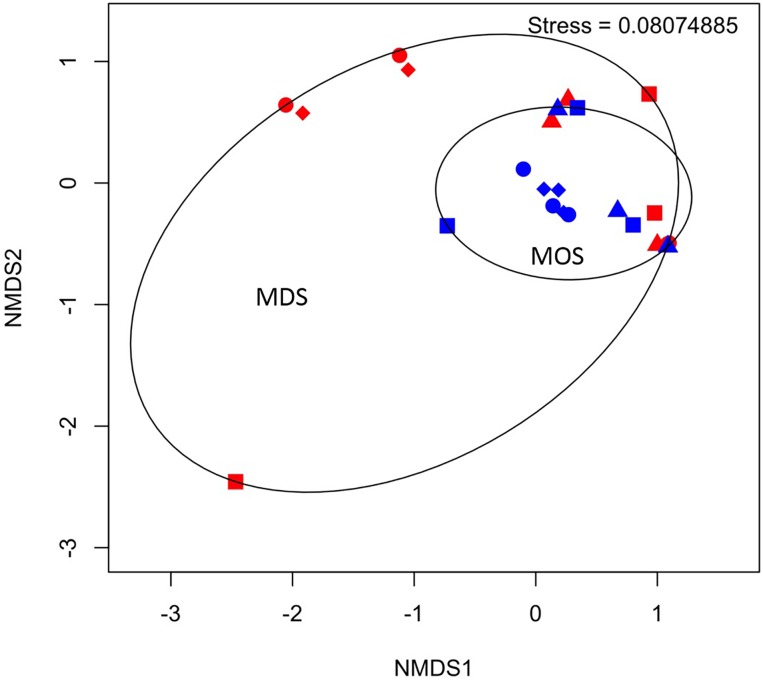
Non-metric multidimensional scaling (NMDS) of bacterial communities associated with aggregates of MDS (red symbols) and MOS (blue symbols) at week 1 (T1; circles), week 2 (T2; triangles), week 3 (T3; diamonds), and week 4 (T4; squares).

Barcoded 16S rRNA Illumina MiSeq technology was employed to study the bacterial community associated with MOS and, for the first time, with MDS over the duration of these experiments. Triplicates of MOS and MDS aggregates were sampled at each time point (T1, T2, T3, and T4), and demonstrated the bacterial composition at family level classification (Figure 4). We did not identify any significant difference in the bacterial community composition between the MOS and MDS aggregates (PERMANOVA, *F*_24_ = 1.12, *p* = 0.265), suggesting that the oil and dispersant, which were solely associated with MOS from the CEWAF or CEWAF+N treatments, had no significant influence in structuring the community of these aggregates. However, an analysis of the similarity between these two types of aggregates at each time point revealed that MDS aggregates constitute a somewhat more diverse bacterial community than the community associated with MOS, although the difference in bacterial community composition between MDS and MOS aggregates was not significant (*p* = 0.215) (Figure 5). Using rarefaction to examine the alpha-diversity of the two aggregate types indicates that MDS aggregates have a more diverse community overall than the MOS aggregate communities (Supplementary Figure S1).

Both MOS and MDS aggregates were dominated by members of the family *Alcanivoraceae*, *Alteromonadaceae*, and *Rhodobacteraceae*, with percentage compositions ranging from 10% to as high as 95% of the total bacterial community composition associated with these aggregates. These taxa appear to be dominant on MOS aggregates across seasons of the year, including for members of the *Pseudoalteromonadaceae*, on MOS aggregates formed in roller bottles with seawater collected from the FSC during the winter of 2015 (Suja et al., 2017). We note that even between replicates of MOS or MDS aggregates that were sampled from the same treatment, the community composition for some showed variability. For example, two of the three replicates of MDS aggregates derived from the SW+D treatment at week 2 were identified with *Kiloniellaceae* as a major group; similarly, this was a dominant group associated with other MDS and MOS aggregates, but not with all the replicates from the same treatment. Other major groups identified, but that were not consistently identified in respective replicates from the same treatment, were *Kiloniellales*, *Flavobacteriaceae*, and *Methylococcales*. We attribute this inconsistency between replicates as an indication that not all aggregates (for MDS or MOS) have the same community composition, even for aggregates sampled from the same roller bottle treatment. This microbial community patchiness has also been described for seawater at the microscale level (Azam, 1998; Fuhrman, 2009) and here, we show it to occur on MDS and MOS aggregates.

Based on the rarefaction curves and 3D-NMDS plots (Figure 5 and Supplementary Figure S1), the phylogenetic diversity (Faith, 1992) of the MDS aggregates appeared greater than that of the MOS aggregates. The former thus represented a higher alpha-diversity of bacterial composition. This also shows that the sequencing depth employed was sufficient to adequately characterize the bacterial diversity, as both treatment types reached an asymptote by a sequencing depth of 10,000. However, this did not result in a statistically significant difference in the *alpha*-diversity between the two treatments (H’), at either the family level (ANOVA, *F*_22_ = 0.486, *p* = 0.493) or SNVs (ANOVA, *F*_22_ = 0.335, *p* = 0.569). The bacterial communities of both the MOS and MDS aggregates were, hence, not significantly different.

**FIGURE 6:**
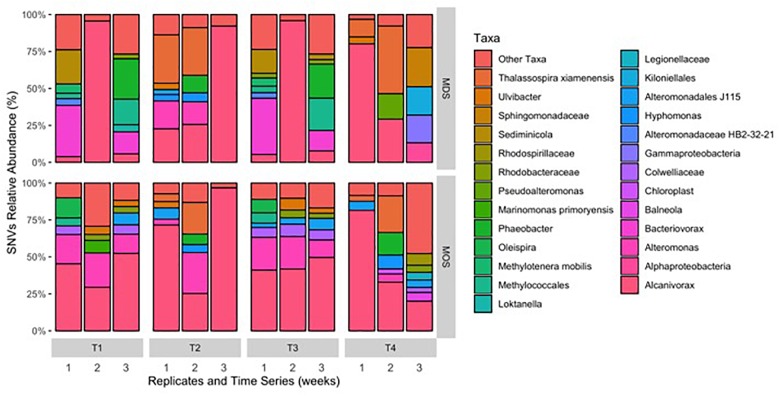
Single nucleotide variant (SNV) relative abundance classification of bacterial 16S rRNA gene MiSeq reads for MDS and MOS aggregates at week 1 (T1), 2 (T2), 3 (T3), and 4 (T4) from treatments SW+D and CEWAF+N, respectively. Sequences were classified to genus-level taxonomy when possible and otherwise at higher level classification is shown.

As both MDS and MOS aggregates harbor a similar bacterial community, dominated by members of the family *Alcanivoraceae*. Further classification down to the genus level revealed they were dominated by members of the genus *Alcanivorax*, with minor representation by *Oleispira* (Figure 6) – organisms which are commonly found enriched at oil-contaminated sites and recognized in playing an important role in the biodegradation of oil hydrocarbons in marine environments (Head et al., 2006; Yakimov et al., 2007). Generalist hydrocarbonoclastic organisms that were also found associated with the MDS and MOS aggregates included *Pseudoalteromonas* and *Alteromonas*, and whilst these organisms would be expected to contribute a role in the biodegradation of the oil associated with, or immediate vicinity of, the aggregates, these organisms are also commonly associated with producing EPS (Arias et al., 2003; Mancuso Nichols et al., 2004; Bhaskar and Bhosle, 2005; Gutierrez et al., 2007, 2008, 2009, 2013). As such, we posit that these organisms effected a heightened production of EPS that was observed under conditions that yielded MDS and MOS (SW+D, CEWAF, CEWAF+N). We further posit that, since the bacterial communities associated with these two types of aggregates were similar, these communities may comprise taxa, such as *Colwellia*, with the capability to utilize the dispersant as a carbon source. We found members of this genus as a major group in some of the MDS and MOS aggregates from, respectively, the SW+D and CEWAF+N treatments. Supporting this, Kleindienst et al. (2015) showed potential dispersant-degrading *Colwellia* selected for in only roller bottle experiments amended with the chemical dispersant Corexit, which also bloomed during the DwH oil spill in the Gulf of Mexico.

## Conclusion

As documented for the DwH, Ixtoc-I and *Tsesis* oil spills, in the event of a spill at sea the formation of MOS is an important process leading to the transportation of the oil from the upper water column to the seafloor. Our study shows that EPS concentrations in seawater become significantly higher as a response to when a chemical dispersant is applied, and interestingly this occurred irrespective of whether crude oil is present or not. Whilst we observed this for surface seawaters of the subarctic northeast Atlantic, future work should assess whether this dispersant-induced response in EPS production would also occur in other ocean regions. This response is likely conferred by EPS-producing bacteria, and while it has only been described in this and one other study, we posit that it is a key modality in the formation of MOS when chemical dispersants are used. Our study shows that in the event of an oil spill in the FSC, the use of dispersants would likely lead to the formation of MOS and MDS, and that both these types of aggregates harbor a similar bacterial community dominated by hydrocarbon degrading and EPS producing bacteria. Although the formation of MDS in the absence of crude oil has been observed in laboratory-based experiments, its significance during the application of chemical dispersants in the event of a spill at sea should not be discounted, and future investigations in this respect should consider MDS formation, including its fate and impacts.

## Data Availability

The datasets generated for this study can be found in SRA repository, SAMN10417120.

## Author Contributions

LS and TG contributed to the design of the work and its interpretation. LS, XC, and SS produced all of the data and together with TG and DP, wrote the manuscript.

## Conflict of Interest Statement

The authors declare that the research was conducted in the absence of any commercial or financial relationships that could be construed as a potential conflict of interest.

## Supplementary Material

FIGURE S1 Rarefaction curves of observed taxa between MOS and MDS aggregates.
