# Supplementary figures and images for: Chemical Dispersant Enhances Microbial Exopolymer (EPS) Production and Formation of Marine Oil/Dispersant Snow in Surface Waters of the Subarctic Northeast Atlantic
Source: Front Microbiol. 2019 Mar 20;10:553. doi: 10.3389/fmicb.2019.00553 (PMC6435573; doi:10.3389/fmicb.2019.00553)

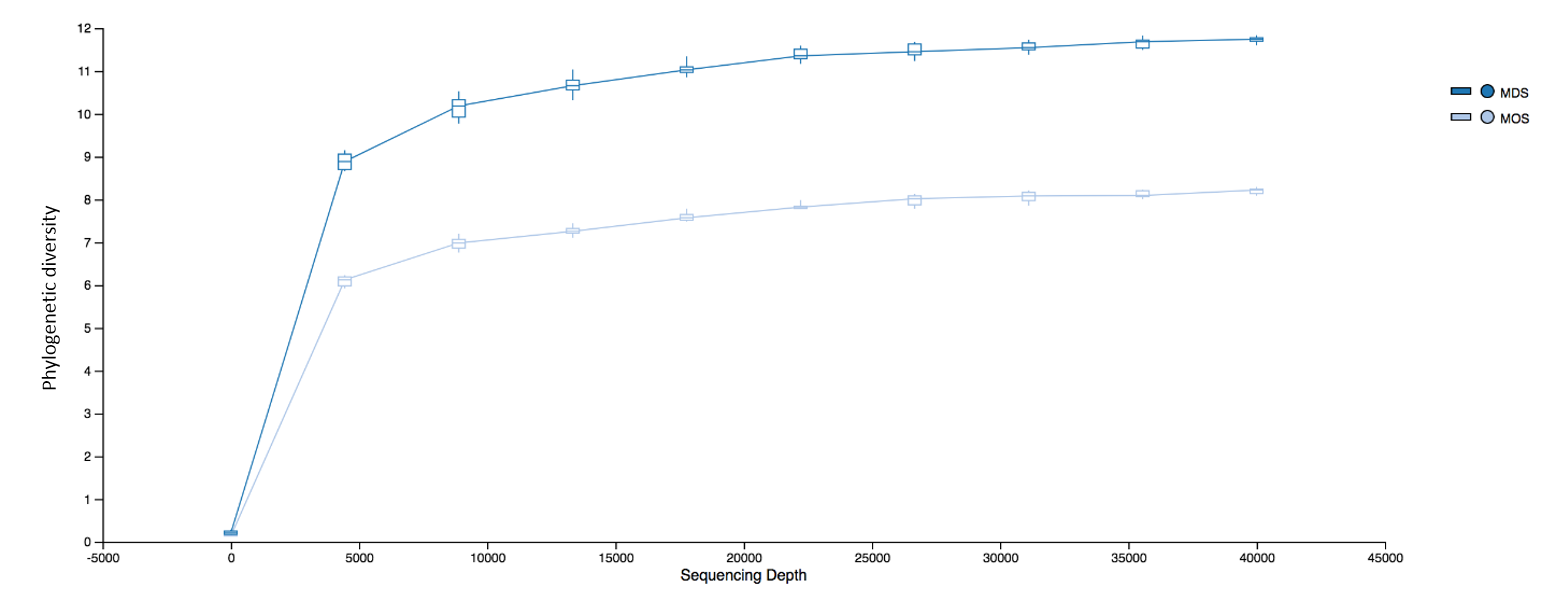

Supplement: FIGURE S1 — Rarefaction curves of observed taxa between MOS and MDS aggregates. [file Image_1.PNG]
